# Logistic Regression Analysis of the Factors Involved in the Failure of Osseointegration and Survival of Dental Implants with an Internal Connection and Machined Collar: A 6-Year Retrospective Cohort Study

**DOI:** 10.1155/2021/9684511

**Published:** 2021-10-07

**Authors:** Aritza Brizuela-Velasco, Ángel Álvarez-Arenal, Esteban Pérez-Pevida, Iker Bellanco-De La Pinta, Héctor De Llanos-Lanchares, Ignacio González-González, Carolina Larrazábal-Morón

**Affiliations:** ^1^Department of Surgery and Medical-Surgical Specialties, Faculty of Medicine, University of Oviedo, Oviedo, Spain; ^2^Department of Surgery, Faculty of Medicine, University of Salamanca, Salamanca, Spain; ^3^Faculty of Health Sciences, Miguel de Cervantes European University, Valladolid, Spain; ^4^School of Dentistry, Catholic University San Vicente Martir of Valencia, Valencia, Spain

## Abstract

**Background:**

Although the long-term success rate of dental implants is currently close to 95%, it is necessary to provide more evidence on the factors related to the failure of osseointegration and survival.

**Purpose:**

To establish the risk factors associated with the failure of osseointegration and survival of dental implants with an internal connection and machined collar and to establish a predictive statistical model.

**Materials and Methods:**

An analytical, retrospective, and observational clinical study of a sample of 297 implants with a follow-up of up to 76 months. Independent variables related to the implant, patient, and surgical and rehabilitative procedures were identified. The dependent variables were failure of osseointegration and failure of implant survival after prosthetic loading. A survival analysis was carried out by applying the Kaplan-Meier model (significance for *p* < 0.05). The log-rank test and the Cox regression analysis were applied to the factors that presented differences. Finally, the regression logit function was used to determine whether it is possible to predict the risk of implant failure according to the analyzed variables with the data obtained in this study.

**Results:**

The percentages of osseointegration and survival were 97.6 and 97.2%, respectively. For osseointegration, there were significant differences according to gender (*p* = 0.048), and the risk of nonosseointegration was 85% lower in women. Regarding survival, the Cox analysis converged on only two factors, which were smoking and treatment with anticoagulant drugs. The risk of loss was multiplied by 18.3 for patients smoking more than 10 cigarettes per day and by 28.2 for patients treated with anticoagulants.

**Conclusions:**

The indicated risk factors should be considered, but the analysis of the results is not sufficient to create a predictive model.

## 1. Introduction

Although the rehabilitation of edentulism using dental implants has a high predictability, there are many factors that influence its prognosis [1]. These factors are related to the patient, the surgical approach or applied load, the implant used in each case or the final prosthetic rehabilitation, and can limit or have a negative impact on osseointegration or on implant survival [2–4].

Implant failure is defined as the total failure of the implant to fulfill its purpose (functional, aesthetic, or phonetic) due to biological or mechanical causes [5]. It can occur during or after the process of osseointegration, once the definitive prosthetic loading has been carried out and the implant is functioning. Two types of dependent variables are frequently assessed during the second period: implant survival, which exclusively indicates the permanence of the implant in the mouth, or implant success, which is based on implant and peri-implant soft tissue parameters [6]. It can be considered that survival indicates the likelihood of the implant to continue performing its function, while success indicates the likelihood that it will do so without causing additional complications.

The scientific literature on this topic describes a series of risk factors associated with the prognosis of implant rehabilitations.

On the one hand, the intrinsic factors of the patient and the presence of certain systemic pathologies, habits, and/or pharmacological treatments, as well as the characteristics of the implant placement area, are apparently the most significant determining factors for dental implant success and survival rates [7–9]. In this regard, the presence of cardiovascular disease, diabetes, osteoporosis, antibone resorption treatments, and radiotherapy can be risk factors. Even so, the health and psychosocial benefits of implant-supported rehabilitation outweigh the risks inherent to the treatment of elderly patients, who are the main beneficiaries of this type of treatment.

The characteristics of the implant and of its prosthetic connection can also play a crucial role in the prognosis; its macroscopic and surface properties can be decisive depending on the case [10–12]. In this regard, clinical studies suggest that internal connection maybe advantageous for marginal bone preservation and, therefore, for implant survival [13].

On the other hand, the use of an appropriate surgical technique depending on the intrinsic characteristics of each case, the adoption of a correct loading protocol, and the effective selection of the final prosthetic rehabilitation, both in terms of design and material, in addition to the used retention, have an impact on the prevention of long-term dental implant failure [14–16]. However, it should be pointed out that the existing evidence in this field is very limited.

Although the long-term success rate of dental implants is currently close to 95% [17], the presence of multiple combinations of risk factors associated with implant loss should be considered to minimize the risk of failure of implant-supported rehabilitations. Finally, it is also necessary to provide more evidence on the factors related to the failure of osseointegration and survival, independently.

Therefore, the objectives of the present analytical retrospective clinical study were to assess the osseointegration and survival rates of a sample of implants with an internal connection and machined collar, to analyze and compare the risk factors associated with implant failure, and to compare the results with those of previously published studies on the topic.

## 2. Material and Methods

In this observational clinical study, we retrospectively analyzed a sample of patients undergoing rehabilitation with dental implants. All implants and prostheses were placed by the same operator (Brizuela-Velasco, A.), and the data on the analyzed variables were obtained from the medical records of the patients, from the day of implant placement and throughout the follow-up period.

In accordance with point 13 of the general principles of the WMA Declaration of Helsinki (ethical principles for medical research involving human subjects), this observational study does not require to be assessed by an ethics committee. Therefore, our study can be considered a posttrial authorization, in which the assignment of a patient to a specific therapeutic strategy is not decided in advance by the protocol of a trial, rather it is established by the usual practice of dentistry, and the decision to prescribe the procedure is clearly dissociated from the decision to include the patient in the study.

All patients treated from January 2013 to June 2014, with a follow-up of up to 6 years, were included in the study.

Therefore, the inclusion criteria correspond to the indications and contraindications for implant rehabilitation in that sample of patients: Patients who presented partial or total edentulism and who were periodontally healthy with good oral hygiene and without medication-related (taking intravenous bisphosphonates) or systemic (uncontrolled diabetes, immunosuppressed) contraindications.

**Figure 1:**
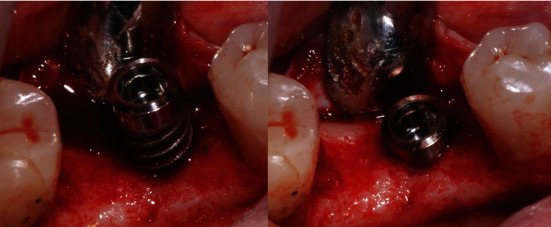
Surgical approach of the insertion of an Klockner Essential Cone implant with a machined collar of 1.5 mm height.

**Figure 2:**
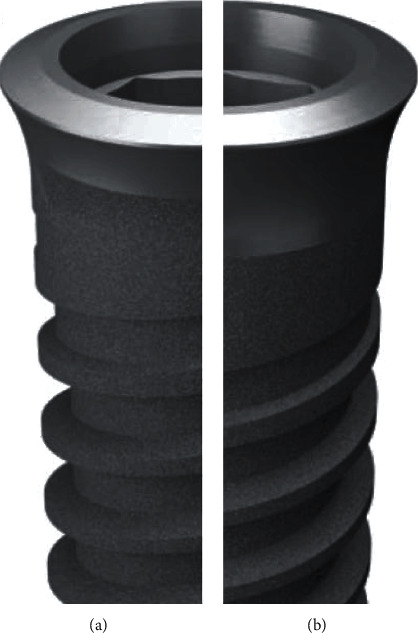
Design of the implants with a machined collar of 0.7 mm (a) and 1.5 (b) height.

All patients were treated with Tissue Level Klockner Essential Cone implants (Klockner Implant System, Madrid, Spain) with an internal connection using a Morse taper and an internal octagon (Figure 1). These implants are made of a commercially pure Titanium Grade 3 with surface modification by blasting alumina particles and acid etching. In their crestal portion, the implants have a machine-polished collar for soft tissue apposition that is available in two heights: 0.7 mm or 1.5 mm (Figure 2). The selection of the height was based on the patient's gingival biotype and aesthetic criteria determined by the clinician (Brizuela-Velasco, A.).

**Figure 3:**
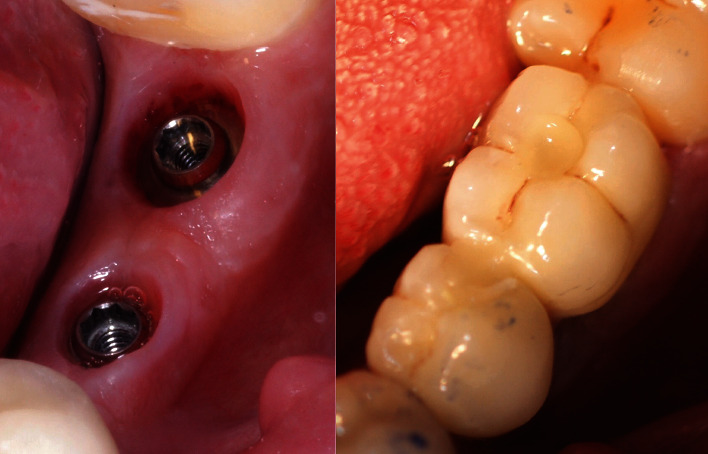
Screw-retained implant-supported prosthetic rehabilitation of two Klockner Essential Cone implants with a machined collar of 1.5 mm height.

All the implants that were assessed and obtained good osseointegration had a follow-up of 59 (minimum) to 76 (maximum) months after implant placement (Figure 3).

The following independent variables were assessed: implant related—length (≤8 mm/10 mm/12 mm), diameter (3.5 mm/4 mm/4.5 mm), and type of machined collar (0.7 mm/1.5 mm); patient related—age, gender (male/female), smoking habits (nonsmoker/smoker of <10 cigarettes per day/smoker of >10 cigarettes per day), previous systemic pathologies (yes/no), anticoagulant drugs (yes/no), arterial hypertension (yes/no), diabetes (yes/no), psychotropic drugs (yes/no), and oral bisphosphonates (yes/no); and related to the surgical and rehabilitative treatment—follow-up time in months, arch (upper/lower), implant position (incisors/canines/premolars/molars), surgical procedure (submerged implants/nonsubmerged implants with transmucosal cap), specific type of surgery (conventional drilling/immediate implant/transcrestal osteotome sinus floor elevation/simultaneous bone regeneration/location of previous regeneration), type of prosthesis (single crown/fixed partial prosthesis/overdenture), and prosthetic retention (cemented/screwed). All these variables were recorded by a single evaluator (Brizuela-Velasco, A.).

The dependent variables analyzed were failure of osseointegration and implant failure after loading (no survival).

The SPSS 25.00 package (IBM SPSS Statistics, New York, USA) was used to carry out the statistical analysis. In addition to a descriptive analysis of the data obtained from each of the assessed variables, a survival analysis was performed by applying the Kaplan-Meier model to obtain the mean curve of survival months, with a 95% confidence interval and a statistical significance when *p* < 0.05. The log-rank test was applied to analyze the significant differences in implant survival for the different studied factors. On the other hand, the Cox regression analysis was used to determine the implant survival risk rate for each factor of the study. The *p* value of the Wald test was 0.05 at the start of the model and 0.1 at the end. Finally, the regression logit function was used to determine whether it is possible to predict the risk of implant failure according to the analyzed variables with the data obtained in this study. This study complies with all checklist items of the statement of the STROBE Initiative (Strengthening the Reporting of Observational Studies in Epidemiology).

## 3. Results

This study assessed 297 implants in 110 patients with a mean age of 56.3 (±11.8). Tables 1 and 2 show the descriptive statistics of osseointegration failures and failures after loading, respectively, for each independent variable analyzed.

With a sample of 297 cases and to achieve a statistical power of 80%, with a significance level of 5%, will be considered as statistically significant an odds ratio of 0.5 when *p*_2_ ≤ *p*_1_ and 1.4 when *p*_2_ ≥ *p*_1_ provided that *P*(*Y* = 1 | *X* = 1) = 0.2, 0.7 when *p*_2_ ≤ *p*_1_ and 1.5 when *p*_2_ ≥ *p*_1_ provided that *P*(*Y* = 1 | *X* = 1) = 0.5, and 0.7 when *p*_2_ ≤ *p*_1_ and 2.7 when *p*_2_ ≥ *p*_1_ provided that *P*(*Y* = 1 | *X* = 1) = 0.7.

The mean follow-up time was 64.5 (±11.7) months. There was osseointegration failure in 7 implants, which represents a success in 97.6% of the placed implants. In turn, 8 implants did not survive after loading, which represents a survival of 97.2% of the osseointegrated implants during the follow-up period.

### 3.1. Analysis of Nonosseointegrated Implants

The Kaplan-Meier model of survival and the log-rank test were applied to find significant differences for the assessed factors. The only factor for which there were significant differences was gender (*p* = 0.048), with a lower survival in men; that is, men had a lower percentage of osseointegration compared to women.

Regarding the Cox regression analysis, as predicted by the Kaplan-Meier model, there was no factor influencing osseointegration except gender, so no Cox model converged.

The logit estimation model showed that the risk of nonosseointegration was reduced by 85% in implants placed in women compared to men (Table 3).

### 3.2. Analysis of Nonsurviving Implants after Loading

The mean implant survival time was 73.4 months. Almost all implant losses occurred before 60 months; the survival was stable between 60 and 70 months of follow-up; finally, there was a slight decrease in survival at 75 months, with definitive survival percentages of approximately 97%.

The log-rank test was applied to compare survival functions to determine whether there were significant differences in implant survival functions for the different factors. It should be noted that this comparison could not be performed for several factors for which there were no losses in any of the categories, such as failure after prosthetic loading, specific type of surgery, type of prosthesis, arterial hypertension, psychotropic drugs, and bisphosphonates.

There were significant differences in survival for the factors: gender, smoking, and anticoagulant drugs (Table 2).

The mean implant survival was 70.5 months in men and 75.3 months in women, and this was statistically different (*p* ≤ 0.001).

The mean survival time was 73.9, 71.6, and 68.9 months for nonsmokers, smokers of less than 10 cigarettes, and smokers of more than 10 cigarettes, respectively, with statistically significant differences (*p* = 0.049).

The mean implant survival period was 74.3 months in patients who were not on anticoagulant drugs and 66.4 for those who were, and this difference was statistically significant (*p* = 0.002).

The Cox regression analysis was used to analyze the survival risk rate for the different analyzed factors. The Cox analysis requires establishing a reference category for each factor, since it compares by how much the impact or risk rate is multiplied if the implant belongs to a given category with respect to the reference. In the case of this study, the first category of each factor was considered as the reference.

After performing the Cox analysis, the model converged on only two factors, smoking and treatment with anticoagulant drugs. This means that these variables produce significant differences in the survival risk rate or loss rate (Table 4).

In this regard, the survival time decreased by 4.2% in patients who smoked more than 10 cigarettes per day when compared with those who did not smoke.

On the other hand, the survival time decreased by 4.4% in patients on anticoagulants when compared with those not on these drugs.

Finally, the logit estimation model could detect those predictors that influence the risk of implant loss and their impact has been quantified.

It was thus determined that the factors that influenced survival also predicted the risk of implant loss; it was multiplied by 18.3 for patients who smoked more than 10 cigarettes per day and by 28.2 for patients on anticoagulants.

The logistic regression models also provided a classification or prognosis of the implants (whether they will be lost or not) based on the estimated probability. To do this, a probability 0.1 was selected as the cut-off point that provides the optimum correction percentages for the study. Any implant with a probability of >0.1 was classified as lost.

The results demonstrated that our study adequately classified the implants, both intact and lost (sensitivity and specificity). However, it was not a good predictive model for loss since 88.5% of the implants diagnosed as lost were found to be intact. Therefore, it is necessary to look for more potential factors of implant loss to establish a predictive model.

## 4. Discussion

The objective of this retrospective clinical study was to assess the success of osseointegration, the survival of implants with an internal connection and machined collar, and to analyze the impact of risk factors associated with implant failure.

The study included a sample of 297 implants, and osseointegration failure occurred in 7 cases, which represents an osseointegration success rate of 97.6%. These results are similar to those of previous studies, which reported that implants have an osseointegration success rate of 71.4 to 98.7% depending on their location within the arches [18].

On the other hand, 8 implants of the final sample of 290 implants assessed after loading failed, which represents a survival of 97.2% during the follow-up period of up to 76 months. These results agree with the findings of a systematic review of the literature that included 23 studies and 7711 implants and revealed that implant survival rate in a 10-year follow-up is approximately 95% [19].

To meet the objectives of this study, an inferential statistic was performed to compare and establish the probability of osseointegration failure and survival for the dependent variables related to the implant, patient, and procedure.

### 4.1. Variables Related to the Implant

The log rank test found no differences in osseointegration and survival for the following implant variables: length, diameter, and height of the machined collar. However, for osseointegration, due to the proximity of the *p* value to the acceptance threshold (*p* = 0.091), there was a trend for osseointegration to be greater in lengths of 10 mm compared to the other lengths.

It is believed that the length of the implant can affect osseointegration since it can be correlated with the possibility of obtaining a greater primary stability. On the other hand, the implant diameter and the height of the machined collar should be more relevant during loading, one for determining the possibilities of stress dissipation and the other for its relationship with the maintenance of a correct biological space. However, the meta-analyses that assessed the relationship of the length and diameter of the implant with its survival did not find statistically significant differences [20, 21]. Similarly, there are no survival studies in the literature that compare different heights of the machined collar, although there are controlled and randomized clinical trials that show similar results for both marginal bone loss and long-term prognosis [22].

### 4.2. Variables Related to the Patient

There were only three factors in the present study that showed statistically significant differences regarding their influence on implant survival. Furthermore, two of them, smoking and anticoagulants, were indicative of a higher risk of implant failure.

As for smoking, there was a decrease of 4.2% in the survival rate of patients who smoked more than 10 cigarettes per day and the risk of implant failure was multiplied by 18.3. Similar results are found in most of the survival studies analyzed in systematic reviews and meta-analyses, which conclude that smoking significantly reduces long-term success and survival rates [23–25]. This is mainly due to the vasoconstrictor properties of tobacco, which hinder proper vascularization and clot formation in the initial phases of osseointegration. Furthermore, smoking is usually associated with a lower level of oral hygiene, thus increasing the risk of dental implant failure [25].

The implants placed in patients on anticoagulants showed a 4.4% decrease in the survival rate, and the risk of implant loss was multiplied by 28.2. The implant survival studies that analyzed the administration of oral anticoagulants as a risk factor for failure did not find significant differences between patients with and without cardiovascular disease. However, there is a higher percentage of implant loss in anticoagulated patients. According to these studies, the administration of anticoagulants does not seem to influence survival by itself, but it is a potential risk factor, particularly in elderly patients with chronic systemic pathologies and long-term pharmacological treatments [7, 26]. This finding agrees with the results obtained in our study, since all patients treated with oral anticoagulants suffered from some other systemic pathology and were older than the mean age. Therefore, age and comorbidities seem to be stronger determining factors for implant success and survival than the administration of specific drugs; this may be attributed to the lack of autonomy and insufficient oral care in older and comorbid patients [7].

The last factor influencing implant survival time was gender. With a survival of 70.5 months in men and 75.3 in women, the difference was significant. Nevertheless, it was not established as a risk factor for implant failure. These results are consistent with those of several retrospective clinical studies that show a higher short- and long-term implant failure rate for men compared to women [27, 28] and can be attributed to a higher consumption of tobacco and less concern for oral hygiene in men compared to women, although the evidence in this regard is limited.

Finally, there was no osseointegration in 7 implants of the present study before prosthetic rehabilitation, and these were independently analyzed to assess potential risk factors for this event. The only factor showing significant differences in survival was gender; osseointegration was statistically lower in men than in women. Similar retrospective studies agree with these results, showing a higher rate of early implant failure in men [29].

However, the regression logit function revealed that it is not possible to develop a predictive model with the data obtained in this retrospective clinical study.

### 4.3. Variables Related to the Surgical and Rehabilitative Procedure

Again, the log rank test found no differences in osseointegration and survival related to the different variables of the surgical or rehabilitative procedure. However, some clinical studies observe important differences in osseointegration related to the position in the arch, namely, the study of Drago [18] that shows values ranging from 71.4% in the posterior maxilla to 98.7% in the anterior mandible. The surgical placement protocol (one-stage vs. two-stage) deserves a special mention. A 2009 meta-analysis concluded that no statistically significant differences were observed between the two procedures; however, trends suggested less implant failures with the 2-stage (submerged) approach especially in fully edentulous patients [30]. Finally, another controversial factor related to implant survival is the type of retention: screwed or cemented. The 2015 meta-analysis of Lemos et al. found higher survival rates and lower marginal bone loss for cemented prostheses and higher prosthetic complications for screwed prostheses. However, the authors considered that the differences were not clinically significant [31].

## 5. Conclusions

Considering the intrinsic limitations of this study, the following conclusions can be drawn:

- The internal connection and machined collar implants have a high rate of osseointegration (97.6%) and survival (97.2%) in a follow-up period of up to 76 months
- The male gender is associated with more osseointegration failures
- The male gender, smoking more than 10 cigarettes per day, and anticoagulant treatment seem to influence survival after loading of dental implants
- These conclusions should be considered with caution since the results are not sufficient to develop a predictive model and it is necessary to look for more potential factors responsible for failure

## Figures and Tables

**Table 1:** Descriptive analysis of the failure of osseointegration in order to the analyzed independent variables and survival analysis applying the Kaplan-Meier model.

		Osseointegration						
		Total		Yes		No		Kaplan-Meier
		Count	Column (*N*, %)	Count	Column (*N*, %)	Count	Column (*N*, %)	^∗^Sig. *p* < 0.05
Sex	Total	297	100.0%	290	100.0%	7	100.0%	^∗^0.048
	Male	145	48.8%	139	47.9%	6	85.7%	
	Female	152	51.2%	151	52.1%	1	14.3%	
Dental arch	Total	297	100.0%	290	100.0%	7	100.0%	0.546
	Upper	160	53.9%	157	54.1%	3	42.9%	
	Lower	137	46.1%	133	45.9%	4	57.1%	
Implant position in the dental arch	Total	297	100.0%	290	100.0%	7	100.0%	0.070
	Incisive	39	13.1%	38	13.1%	1	14.3%	
	Canine	35	11.8%	32	11.0%	3	42.9%	
	Premolar	101	34.0%	100	34.5%	1	14.3%	
	Molar	122	41.1%	120	41.4%	2	28.6%	
Implant length	Total	297	100.0%	290	100.0%	7	100.0%	0.091
	6.0	12	4.0%	12	4.1%	0	0.0%	
	8.0	109	36.7%	104	35.9%	5	71.4%	
	10.0	118	39.7%	118	40.7%	0	0.0%	
	12.0	58	19.5%	56	19.3%	2	28.6%	
Implant diameter	Total	297	100.0%	290	100.0%	7	100.0%	0.854
	3.5	98	33.0%	95	32.8%	3	42.9%	
	4.0	145	48.8%	142	49.0%	3	42.9%	
	4.5	54	18.2%	53	18.3%	1	14.3%	
Smooth polished neck height	Total	297	100.0%	290	100.0%	7	100.0%	0.963
	0.7 mm	173	58.2%	169	58.3%	4	57.1%	
	1.5 mm	124	41.8%	121	41.7%	3	42.9%	
Surgical technique	Total	297	100.0%	290	100.0%	7	100.0%	0.633
	Conventional drilling mature bone	219	73.7%	214	73.8%	5	71.4%	
	Postdental exodontia	36	12.1%	34	11.7%	2	28.6%	
	Atraumatic sinus lift	17	5.7%	17	5.9%	0	0.0%	
	Simultaneous bone regeneration	20	6.7%	20	6.9%	0	0.0%	
	Located in previously regenerated bone	5	1.7%	5	1.7%	0	0.0%	
Surgical phases	Total	297	100.0%	290	100.0%	7	100.0%	0.479
	One phase	173	58.2%	168	57.9%	5	71.4%	
	Two phases	124	41.8%	122	42.1%	2	28.6%	
Smoker	Total	297	100.0%	290	100.0%	7	100.0%	0.242
	No	231	77.8%	227	78.3%	4	57.1%	
	<10 cig/day	36	12.1%	35	12.1%	1	14.3%	
	>10 cig/day	30	10.1%	28	9.7%	2	28.6%	
Previous medical conditions	Total	297	100.0%	290	100.0%	7	100.0%	0.498
	No	204	68.7%	200	69.0%	4	57.1%	
	Yes	93	31.3%	90	31.0%	3	42.9%	
Anticoagulant drugs	Total	297	100.0%	290	100.0%	7	100.0%	0.327
	No	251	84.5%	246	84.8%	5	71.4%	
	Yes	46	15.5%	44	15.2%	2	28.6%	
Arterial hypertension	Total	297	100.0%	290	100.0%	7	100.0%	0.419
	No	272	91.6%	265	91.4%	7	100.0%	
	Yes	25	8.4%	25	8.6%	0	0.0%	
Diabetes	Total	297	100.0%	290	100.0%	7	100.0%	0.126
	No	286	96.3%	280	96.6%	6	85.7%	
	Yes	11	3.7%	10	3.4%	1	14.3%	
Psychoactive drugs	Total	297	100.0%	290	100.0%	7	100.0%	0.419
	No	272	91.6%	265	91.4%	7	100.0%	
	Yes	25	8.4%	25	8.6%	0	0.0%	
Bisphosphonate drugs	Total	297	100.0%	290	100.0%	7	100.0%	0.702
	No	291	98.0%	284	97.9%	7	100.0%	
	Yes	6	2.0%	6	2.1%	0	0.0%	

**Table 2:** Descriptive analysis of the failure of survival (after loading) in order to the analyzed independent variables and survival analysis applying the Kaplan-Meier model.

		Survival						
		Total		Yes		No		Kaplan-Meier
		Count	Column (*N*, %)	Count	Column (*N*, %)	Count	Column (*N*, %)	^∗^Sig. *p* < 0.05
Sex	Total	290	100.0%	282	100.0%	8	100.0%	^∗^0.05
	Male	139	47.9%	132	46.8%	7	87.5%	
	Female	151	52.1%	150	53.2%	1	12.5%	
Dental arch	Total	290	100.0%	282	100.0%	8	100.0%	0.282
	Upper	157	54.1%	154	54.6%	3	37.5%	
	Lower	133	45.9%	128	45.4%	5	62.5%	
Implant position in the dental arch	Total	290	100.0%	282	100.0%	8	100.0%	0.373
	Incisive	38	13.1%	35	12.4%	3	37.5%	
	Canine	32	11.1%	32	11.3%	0	0.0%	
	Premolar	100	34.4%	98	34.7%	2	25	
	Molar	120	41.4%	117	41.6%	3	37.5	
Implant length	Total	290	100.0%	282	100.0%	8	100.0%	0.870
	6.0	12	4.2%	12	4.3%	0	0.0%	
	8.0	104	35.8%	100	35.5%	4	50%	
	10.0	118	40.6%	116	41.1%	2	25%	
	12.0	56	19.4%	54	19.1%	2	25%	
Implant diameter	Total	290	100.0%	282	100.0%	8	100.0%	0.830
	3.5	95	32.7%	90	31.9%	5	62.5%	
	4.0	142	49.0%	142	50.4%	0	0.0%	
	4.5	53	18.3%	50	17.7%	3	37.5%	
Smooth polished neck height	Total	290	100.0%	282	100.0%	8	100.0%	0.582
	0.7 mm	169	58.2%	163	57.8%	6	75%	
	1.5 mm	121	41.8%	119	42.2%	2	25%	
Surgical technique	Total	290	100.0%	282	100.0%	8	100.0%	0.272
	Conventional drilling mature bone	214	73.8%	210	74.1%	4	50%	
	Postdental exodontia	34	11.7%	33	11.6%	3	37.5%	
	Atraumatic sinus lift	17	5.9%	17	6.0%	0	0.0%	
	Simultaneous bone regeneration	20	6.9%	19	6.6%	1	12.5%	
	Located in previously regenerated bone	5	1.7%	5	1.7%	0	0.0%	
Surgical phases	Total	290	100.0%	282	100.0%	8	100.0%	0.563
	One phase	168	57.9%	165	58.5%	3	37.5%	
	Two phases	122	42.1	117	41.5%	5	62.5%	
Smoker	Total	290	100.0%	282	100.0%	8	100.0%	^∗^0.049
	No	227	78.3%	222	78.7%	5	62.5%	
	<10 cig/day	35	12.1%	34	12.0%	1	12.5%	
	>10 cig/day	28	9.6%	26	9.3%	2	25%	
Previous medical conditions	Total	290	100.0%	282	100.0%	8	100.0%	0.093
	No	200	69.00	197	69.8%	3	37.5%	
	Yes	90	31.0%	85	30.2%	5	62.5%	
Anticoagulant drugs	Total	290	100.0%	282	100.0%	8	100.0%	^∗^0.002
	No	246	84.8%	243	86.2%	3	37.5%	
	Yes	44	15.2%	39	13.8%	5	62.5%	
Arterial hypertension	Total	290	100.0%	282	100.0%	8	100.0%	0.268
	No	265	91.4%	257	91.1%	8	100.0%	
	Yes	25	8.6%	25	8.9%	0	0.0%	
Diabetes	Total	290	100.0%	282	100.0%	8	100.0%	0.799
	No	280	96.5%	272	96.4%	8	100.0%	
	Yes	10	3.5%	10	3.6%	0	14.3%	
Psychoactive drugs	Total	290	100.0%	290	100.0%	8	100.0%	0.231
	No	265	91.4%	257	91.1%	8	100.0%	
	Yes	25	8.6%	25	8.9%	0	0.0%	
Bisphosphonate drugs	Total	290	100.0%	282	100.0%	8	100.0%	0.600
	No	284	97.9%	276	97.9%	8	100.0%	
	Yes	6	2.1%	6	2.1%	0	0.0%	
Prosthesis retention system	Total	240	100.0%	235	100.0%	5	100.0%	0.492
	Cemented	66	27.5%	64	27.2%	2	40.0%	
	Screwed	174	72.5%	171	72.8%	3	60.0%	
Type of prosthesis	Total	290	100.0%	282	100.0%	8	100.0%	0.472
	Single crown	84	29.0%	84	29.8%	0	0.0%	
	Fixed partial prosthesis (FPP)	155	53.4%	150	53.2%	5	62.5%	
	Overdenture	51	17.6%	48	17.0%	3	37.5%	

^∗^Statistically significant findings.

**Table 3:** Results of the logit estimation model for nonosseointegration for risk factor sex, with a 10% significance level (*p* value < 0.1). Odds ratio = *p*/(1 − *p*) = 0.045∗0.149^Sex^.

		*B*	S.E.	Wald	*df*	Sig.	Exp (*B*)	90% CI for Exp (*B*)	
								Lower	Upper
Step 1^a^	Sex: female	-1.905	1.087	3.072	1	0.080	0.149	0.025	0.889
	Constant	-3.106	0.417	55.406	1	0.000	0.045		

**Table 4:** Cox regression analysis for the dependent variable failure of survival.

		*B*	SE	Wald	*df*	Sig.	Exp (*B*)	95.0% CI for Exp (*B*)	
								Lower	Upper
Pass 1	Anticoagulant: yes (cat. ref. no)	2.050	0.742	7.621	1	0.006	7.764	1.812	33.268
Pass 2	Smoker: no (cat. ref.)			5.747	2	0.056			
	Smoker: <10 cig/day	1.022	1.138	0.807	1	0.369	2.780	0.299	25.843
	Smoker: >10 cig/day	3.117	1.302	5.729	1	^∗^0.017	22.585	1.759	289.996
	Anticoagulant	3.170	1.124	7.957	1	^∗^0.005	23.814	2.632	215.508

*H*(*t*) = *H*0(*t*)∗22.585^>10 cig/day^∗23.814^Anticoagulant^. ^∗^Statistically significant findings.

## Data Availability

The data could be provided under request.

## Additional Points

*Summary Box*. What is known: Long-term success rate of dental implants is close to 95%; risk factors associated with implant loss should be considered. It is also necessary to provide more evidence on the factors related to the failure of osseointegration and survival. What this study adds: This study ratifies the most prevalent risk factors related to implant failure and underlines the significance of those factors related to the patient.
